# Antibiotic exposure in culture-negative preterm infants: a 10-year single-centre study

**DOI:** 10.1038/s41390-025-04707-5

**Published:** 2026-01-10

**Authors:** Cheryl Anne Mackay, Elizabeth A. Nathan, Michelle Claire Porter, Damber Shrestha, Rolland Kohan, Tobias Strunk

**Affiliations:** 1 Neonatology, Child and Adolescent Health Service, King Edward Memorial Hospital, Subiaco, WA Australia; 2 Division of Paediatrics, School of Medicine, University of Western Australia, Crawley, WA Australia; 3 Division of Obstetrics and Gynaecology, School of Medicine, University of Western Australia, Crawley, WA Australia; 4 Microbiology Department, PathWest Laboratory Services, Nedlands, WA Australia; 5 Wesfarmers’ Centre for Vaccines and Infectious Diseases, The Kids Research Institute, Nedlands, WA Australia

## Abstract

**Background:**

Antibiotic exposure in neonatal intensive care units (NICU) is high. This study describes antibiotic use in very preterm infants and examines the association between duration of exposure and outcomes in blood culture negative (CN) infants.

**Methods:**

Infants <32 weeks’ gestation admitted between January 2012 and June 2022 were included in this retrospective cohort study. Data were extracted from electronic databases. Antibiotic exposure was calculated as duration of treatment (DOT) and antibiotic utilisation rate (AUR) and compared with neonatal outcomes including mortality, late onset sepsis (LOS), necrotising enterocolitis (NEC), chronic lung disease (CLD), severe retinopathy of prematurity (ROP) and/or severe brain injury.

**Results:**

There were 3235 CN infants included in the study; 1601 (49.5%) received antibiotics for ≤ 2 days of which 266 (8.2%) received no antibiotics; 841 (26.0%) received antibiotics for ≥ 5 days. DOT decreased from 78.0 to 61.9 per 1000 and AUR from 0.07 (IQR 0.04–0.11) to 0.05 (IQR 0.03–0.10) from 2012 to 2022. Higher AUR and/or prolonged antibiotic exposure was associated with increased mortality, brain injury, NEC, ROP, LOS, and CLD.

**Conclusion:**

Antibiotics are critical for infants with sepsis but can cause harm in those without. Strategies to reduce antibiotic exposure are needed to improve preterm infant outcomes.

**Impact:**

- Prolonged antibiotic exposure is common in culture-negative, very preterm infants.
- Although antibiotics are critical for infants with culture-positive sepsis, they can cause harm in those who are culture-negative.
- This study adds to the small pool of evidence examining antibiotic use and its association with increased morbidity and mortality in very preterm infants.
- The study findings will impact antibiotic prescribing practices significantly and result in strategies to reduce antibiotic exposure in these at-risk infants.

## Introduction

Antibiotics are the most commonly prescribed medications in neonatal intensive care units (NICU) with approximately 60% of infants in NICUs receiving antibiotics in the first three days of admission.^1,2^ Empiric antibiotic treatment is particularly high in preterm infants and is inversely proportional to gestational age. Approximately 3% of infants ≥ 34 weeks’ gestation receive antibiotics during admission, compared with ~80% of those < 32 weeks’ and ~98% of those < 28 weeks’.^3–5^ Often, prolonged courses of antibiotics are administered despite negative cultures.^5^

Prompt antibiotic therapy is appropriate for infants at increased sepsis risk, and for culture-confirmed sepsis appropriate agent choice and treatment duration are essential. However, antibiotic therapy confers no benefit in culture-negative (CN) infants but has been associated with harm beyond driving antimicrobial resistance. Early and/or prolonged antibiotic exposure disrupts the neonatal microbiome at a critical phase of immune development and is associated with worse outcomes in very preterm infants (death, late-onset sepsis (LOS), necrotizing enterocolitis (NEC), chronic lung disease (CLD) and poor growth).^6–9^ It also has longer-term associations with metabolic, immune-mediated and inflammatory conditions in childhood.^7,10^

The higher risk of systemic infection in very preterm infants, predominantly due to distinct qualities of the immature innate and adaptive immune systems, invasive procedures they require and prolonged hospital stays, is associated with significant morbidity and mortality and may drive initiation and/or prolonged antibiotic therapy.^11^ Against this background, we examined a large single-center cohort of inborn infants < 32 weeks’ gestation to describe temporal trends in antibiotic utilization (including duration of therapy) and to evaluate the association between antibiotic exposure and mortality and major neonatal morbidities (LOS, NEC, CLD, severe retinopathy of prematurity (ROP) and/or severe brain injury) to better inform antibiotic prescribing practices and optimize infant outcomes. Contemporary data addressing these questions are scarce globally and, to our knowledge, unknown in Australian cohorts.

## Methods

### Study Design and Participants

A retrospective cohort study was conducted at King Edward Memorial Hospital (KEMH) in Perth. All infants < 32 weeks gestation admitted to the KEMH NICU between January 1, 2012, and June 30, 2022, were included from birth to discharge or transfer out of the unit, which could occur up to 44 weeks’ corrected for gestational age (CGA). Infants with major congenital anomalies were excluded due to higher sepsis risk and antibiotic exposure.^12^ Infants not born at KEMH and those with blood culture-positive early-onset sepsis (EOS) were excluded from the outset. Those with blood culture-positive LOS, NEC, and/or SIP were excluded from the date of diagnosis, as they would justifiably have been commenced on antibiotics as per best practice. All infants with culture-positive cerebrospinal fluid (CSF) had a positive blood culture with the same organism and were only included once to avoid duplication. Infants with missing data were also excluded.

Maternal, neonatal, and follow-up data were retrieved from a unit-specific electronic neonatal database using Microsoft Access (2010)®. The study is reported according to the Strengthening the Reporting of Observational Studies in Epidemiology for Newborn Infection (STROBE-NI) guideline.^13^ It was approved by the Child and Adolescent Health Service (CAHS) Governance, Evidence, Knowledge and Outcomes (GEKO) Triage Committee (000471).

### Study Setting

KEMH is the only tertiary perinatal centre in Western Australia (WA) and covers a geographical area of more than 2.5 million km^2^ with a total population of 2.4 M–2.8 M during the study period. There were on average 5772 births and 2228 neonatal admissions annually during the study period. The average annual stillbirth, preterm birth and neonatal mortality rates in Western Australia were 6.6/1000 births, ~9% and 1.6/1000 livebirths, and facility-specific (KEMH) rates were 2.5/1000 births, ~12% and 5.1/1000 livebirths respectively at the time.

KEMH is a public hospital that provides comprehensive obstetric care and routinely offers resuscitation and intensive care for infants from 23 weeks’ gestation onwards. Antenatal antibiotics are administered for prevention of neonatal *Streptococcus agalactiae* infection using screening- and risk-based criteria, preterm and/or prolonged rupture of membranes >18 h, and clinical evidence of chorioamnionitis. Recommendations for blood collection for culture include a minimum 0.5 ml in infants <28 weeks’ gestation and 1 ml in infants ≥28 weeks’. Empiric treatment for sepsis in the neonate includes penicillin and gentamycin for EOS and vancomycin and gentamycin for LOS. Quality improvement strategies, such as early introduction and faster advancement of enteral feeds, topical coconut oil for skin integrity, decreased central lines, and infection prevention and control measures, were introduced during the study period but there were no significant changes in diagnostic and treatment practices.

### Definitions of Variables

Data was collected and defined according to the Australia and New Zealand Neonatal Network (ANZNN) guidelines.^14^ Study variables were defined as follows:

(i) *Preeclampsia* was defined according to the Society of Obstetric Medicine of Australia and New Zealand guideline for hypertension in pregnancy at the time.^15^
(ii) *Gestational diabetes* was defined according to the Australasian Diabetes in Pregnancy Society’s Consensus Guideline at the time.^16^
(iii) *Chorioamnionitis* diagnosed based on clinical features of maternal fever ( ≥ 38 °C), clinical signs such as uterine tenderness and/or purulent amniotic fluid, and/or laboratory evidence of infection. Not all patients had histological evidence of chorioamnionitis.
(iv) *Antenatal antibiotics* included any systemic antibiotics administered to the mother within 48 h of birth.
(v) *Antenatal steroids* were considered given if at least one dose was given at least 24 h before birth.
(vi) *GA* was assessed on maternal history and obstetric ultrasound at <20 weeks gestation or, in the absence of these, by clinical assessment as per the Dubowitz and Ballard score.^17^
(vii) *Neonatal antibiotics* included only intravenous antibiotics administered during the neonatal admission.
(viii) *Chronic lung disease (CLD)* was defined as supplemental oxygen requirement at 36 weeks corrected gestational age (CGA)^14^
(ix) *NEC* was diagnosed using Bell’s criteria and only NEC ≥ Stage 2 included.^18^
(x) *Neonatal sepsis* was defined by positive blood or CSF culture with a bacterial or fungal pathogen AND antibiotic treatment ≥5 days. EOS was defined as sepsis occurring at <48 h of life and late-onset sepsis LOS as that occurring ≥48 h after birth.^14^ Typically non-pathogenic organisms (contaminants) were pre-specified and included Coagulase negative *staphylococci* (CONS), *Streptococcus viridans, Streptococcus bovis, Micrococcus* species*, Corynebacterium* and *Bacillus cereus*. These were only considered pathogenic if the patient had CRP > 20 mg/L within 48 hours of the positive blood culture AND received antibiotic treatment for ≥5 days.^14^
(xi) *Severe retinopathy of prematurity (ROP)* included stages 3 to 5; aggressive ROP characterised by posterior location, prominent neovascularisation and plus disease, and rapid progression of neovascularisation; and/or ROP requiring treatment.^14^
(xii) *Severe brain injury* was defined as Grade 3 or 4 intraventricular haemorrhage (IVH) as defined by Papile or cystic periventricular leukomalacia (PVL) detected on cranial ultrasound or MRI brain prior to discharge.^19,20^

### Outcome measures

*Antibiotic exposure* was counted in calendar days regardless of the number of individual agents and doses given on that day. Exposure was described as none, ≤ 2 days, 3 to 4 days, and prolonged ( ≥ 5 days). Consecutive and non-consecutive days of antibiotic exposure were considered in the analysis to differentiate the impact of short courses of empiric antibiotic treatment for possible sepsis, discontinued early in response to negative cultures, compared to prolonged consecutive courses of antibiotics in CN infants. The antibiotic utilisation rate (AUR) was defined as the number of days an infant was exposed to 1 or more antimicrobial agents divided by the total length of hospital stay in days. Duration of treatment (DOT) was calculated as total antibiotic days per 1000 inpatient days. Antimicrobial exposure as prophylaxis for fungal or urinary tract infections was not included.

*Neonatal outcomes* included pre-discharge mortality and/or major neonatal morbidity including LOS, NEC ≥ stage 2, CLD, severe ROP and/or severe brain injury until discharge from the neonatal unit.

### Statistical analysis

Maternal and neonatal characteristics were compared between infants with antibiotic exposure ≤2 days, 3–4 days, and ≥5 days. The prevalence of outcomes for infants without any antibiotic exposure was low and these infants were combined with the ≤2 d exposure reference group for analyses. One-way ANOVA or Kruskal-Wallis tests were used for parametric and non-parametric continuous outcomes, and Chi-square tests for categorical outcomes. Durations of ventilation data, central lines and length of stay were summarised using Kaplan-Meier survival estimates and compared between groups using log-rank tests. Deaths were censored for survival analyses.

Temporal trends associated with annual AUR over the study period were analysed using negative binomial regression due to overdispersion in the data and reported as incidence rate ratios (IRR) and 95% confidence intervals. Associations between AUR, or prolonged antibiotic exposure, and binary neonatal outcomes were analysed using logistic regression and reported as odds ratios and 95% confidence intervals. Length of hospital stay was analysed using Cox proportional hazards modelling with deaths censored in analysis and reported as hazard ratios and 95% confidence intervals. The effect of consecutive compared with non-consecutive antibiotic exposure and neonatal outcomes was analysed. All models were adjusted for gestational age, sex, birthweight z-score and year of birth as these were considered potential confounding factors for neonatal outcomes. All tests were two-sided and *p*-values < 0.05 were considered significant. Stata version 17 (College Station, TX; StatCorp LLC) was used for all analyses.

## Results

### Demographic characteristics

**Fig. 1:**
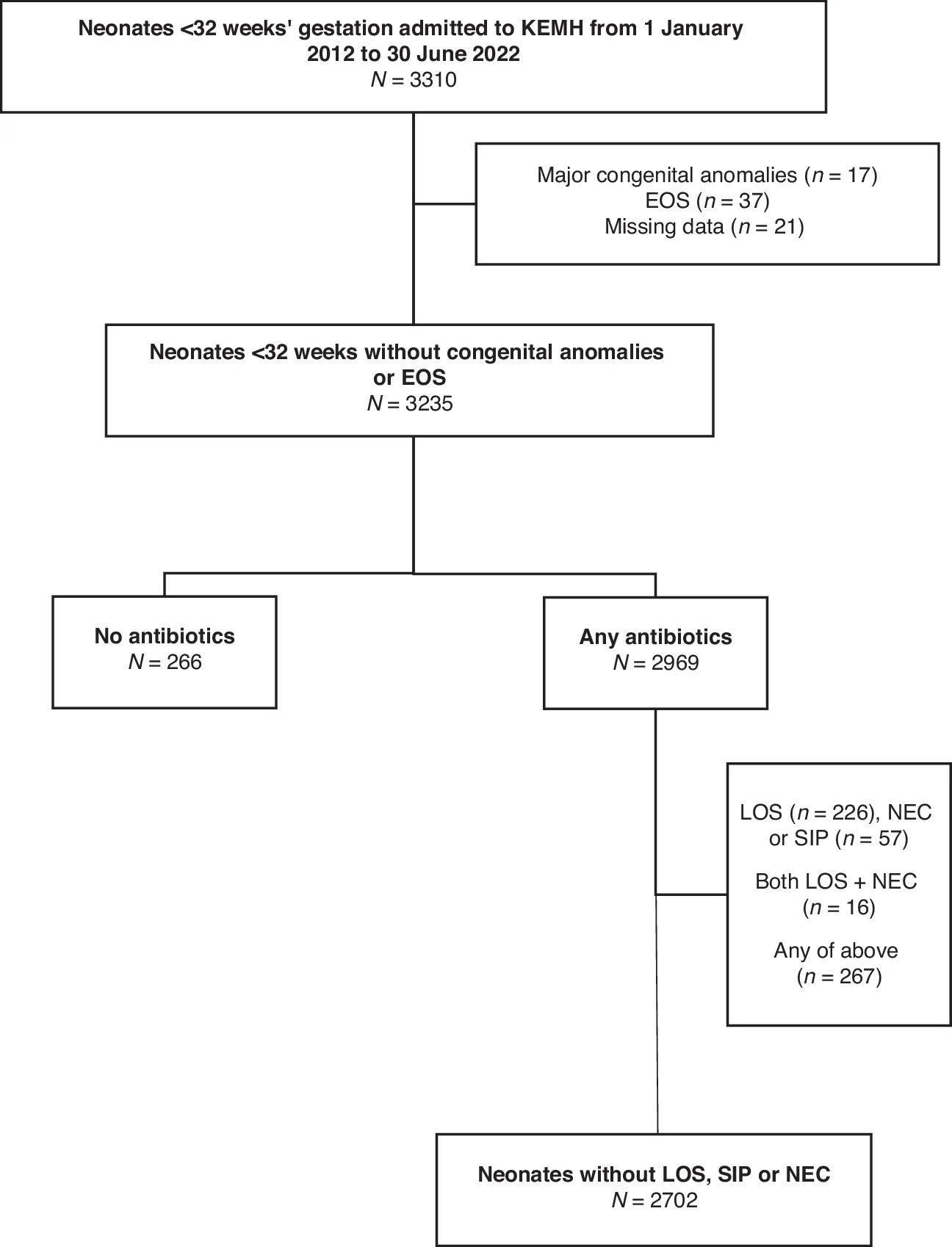
Description of case inclusion in the study. Flow chart describing neonates <32 weeks' gestation who were included in the study and those with major congenital anomalies, early-onset sepsis (EOS), missing data, late-onset sepsis (LOS), necrotising enterocolitis (NEC) and spontaneous intestinal perforation (SIP) who were not included.

There were 3310 neonates <32 weeks gestation admitted to the KEMH NICU during the study period of which 3235 met the inclusion criteria; 2969 infants received antibiotics of any duration, 841 received prolonged antibiotics ( ≥ 5 days), and only 266 remained free of antibiotic exposure. In this cohort, there were 173 deaths, 11 in the first 12 h of life, 86 between 12 h and 7 days and 78 after 7 days of life; 267 infants developed LOS (*n* = 226) and/or NEC/SIP (*n* = 57) and were censored from the date of diagnosis (Fig. 1).

Table 1 describes maternal and neonatal characteristics described according to duration of antibiotic exposure. Mothers of infants exposed to prolonged antibiotics ( ≥ 5 days) were younger, more likely to be Aboriginal or Torres Strait Islander (ATSI), have chorioamnionitis, be treated with antibiotics antenatally, and birth vaginally compared to those with antibiotic exposure ≤2 days. They were less likely to have diabetes and/or multiple births.

**Table 1:** Maternal and neonatal characteristics of infants by days of antibiotic exposure

		Antibiotic exposure (days)			
	All (*n* = 3235) N (%)	≤ 2 N = 1601	3–4 N = 793	≥5 N = 841	*p*^a^
***Maternal characteristics***					
Maternal age (years) (mean, sd)	30.0 (6.0)	30.5 (5.9)	30.1 (5.8)	29.2 (6.2)	< 0.001
Ethnicity (*n*,%)					
Caucasian	2039 (63.0)	1034 (64.6)	509 (64.2)	496 (59.0)	<0.001
ATSI	431 (13.3)	178 (11.1)	103 (13.0)	150 (17.8)	
Other	765 (23.7)	389 (24.3)	181 (22.8)	195 (23.2)	
Preeclampsia (*n*,%)	558 (17.2)	293 (18.3)	130 (16.4)	135 (16.1)	0.288
Gestational diabetes (*n*,%)	365 (11.3)	216 (13.5)	90 (11.3)	59 (7.0)	<0.001
Multiple birth (*n*,%)	891 (27.5)	524 (32.7)	216 (27.2)	151 (18.0)	<0.001
Chorioamnionitis (*n*,%)	423 (13.1)	131 (8.2)	111 (14.0)	181 (21.5)	<0.001
Antenatal antibiotics (*n*,%)	1230 (38.0)	550 (34.4)	314 (39.6)	366 (43.5)	<0.001
Antenatal steroids (*n*,%)	2147 (66.5)	1049 (65.7)	527 (66.6)	571 (68.1)	0.498
Mode of delivery (*n*,%)					
Caesarean section	1979 (61.2)	1031 (64.4)	459 (57.9)	489 (58.1)	<0.001
Vaginal	1251 (38.7)	566 (35.4)	334 (42.1)	351 (41.7)	
Not stated ^b^	4 (0.1)	3 (0.2)	0 (0.0)	1 (0.1)	
Inborn (*n*,%)	2939 (90.9)	1480 (92.4)	695 (87.6)	764 (90.8)	<0.001
***Neonatal characteristics***					
Gestational age (weeks)					
Median (IQR)	29.3 (27.0–30.9)	30.0 (28.3–31.1)	29.3 (27.1–30.9)	27.1 (25.1–29.3)	<0.001
<28 weeks (*n*, %)	1097 (33.9)	347 (21.7)	257 (32.4)	493 (58.6)	<0.001
Birthweight					
Weight (g) (median, IQR)	1205 (907–1518)	1332 (1055–1580)	1210 (945–1528)	916 (710–1260)	<0.001
Z-score (median, IQR)	0.12 (−0.53–0.73)	0.11 (−0.45–0.74)	0.20 (−0.48–0.76)	0.06 (−0.67–0.69)	<0.001
Male sex (*n*, %)	1787 (55.2)	851 (53.2)	442 (55.7)	494 (58.7)	0.029
Apgar<7 at 5 min (*n*, %)	612 (18.9)	233 (14.6)	139 (17.5)	240 (28.6)	<0.001
Ventilation					
Total (*n*, %)	2145 (66.3)	868 (54.2)	548 (69.1)	729 (86.7)	<0.001
Duration (hours) (median, IQR)	24 (10–146)	14 (7–43)	20 (10–89)	129 (21–619)	<0.001
CPAP					
Total (*n*, %)	2977 (92.0)	1446 (90.3)	743 (93.7)	788 (93.7)	0.002
Duration (hours) (mendian, IQR)	249 (67–926)	130 (43–507)	245 (68–914)	906 (290–1250)	<0.001
Central lines					
Total (*n*, %)	1152 (35.6)	331 (20.7)	285 (35.9)	536 (63.7)	< 0.001
Duration (days) (median, IQR)	93 (58–128)	84 (46–111)	83 (46–122)	105 (73–140)	< 0.001

*LOS* late onset sepsis, *NEC* necrotising enterocolitis, *SIP* spontaneous intestinal perforation, *ATSI* Aboriginal or Torres Strait Islander, Antenatal antibiotics included any antibiotics administered within 48 h of birth; Antenatal steroids included at least one dose of steroids ≥24 h before delivery; Ventilation: Any form of invasive ventilation via endotracheal tube, *CPAP* non-invasive continuous positive airway pressure ventilation by nasal prongs.

^a^*p*-value represents the comparison of infants with antibiotic exposure ≤2 d, 3–4 d and ≥5 d; ^b ‘^Not stated’ category was not included in the statistical test.

Neonates exposed to prolonged antibiotics were of lower gestational age and birth weight and were more likely to have a 5-minute Apgar score <7, require longer duration of ventilation and/or CPAP, and have central lines in place during their admission.

### Antibiotic exposure

#### Timing and duration of antibiotic exposure

Overall, DOT was 71.4 per 1000 inpatient days and infants received a median of 2 days antibiotics (IQR 2–5) per patient (Supplementary Table S1A). The majority (2654, 89.4%) were treated for suspected EOS and 689 (23.2%) were treated for suspected LOS (Supplementary Table S1A). There were 1601 (49.5%) infants who received antibiotics for total ≤2 days (ie no antibiotics or <2 days antibiotics), 793 (24.5%) for 3–4 days and 841 (26.0%) for ≥5 days (Table 1). Antibiotic exposure ≥5 days occurred more often in CN (*n* = 749) than culture-positive infants (*n* = 226).

#### Trends in antibiotic exposure over time and by gestational age

**Fig. 2:**
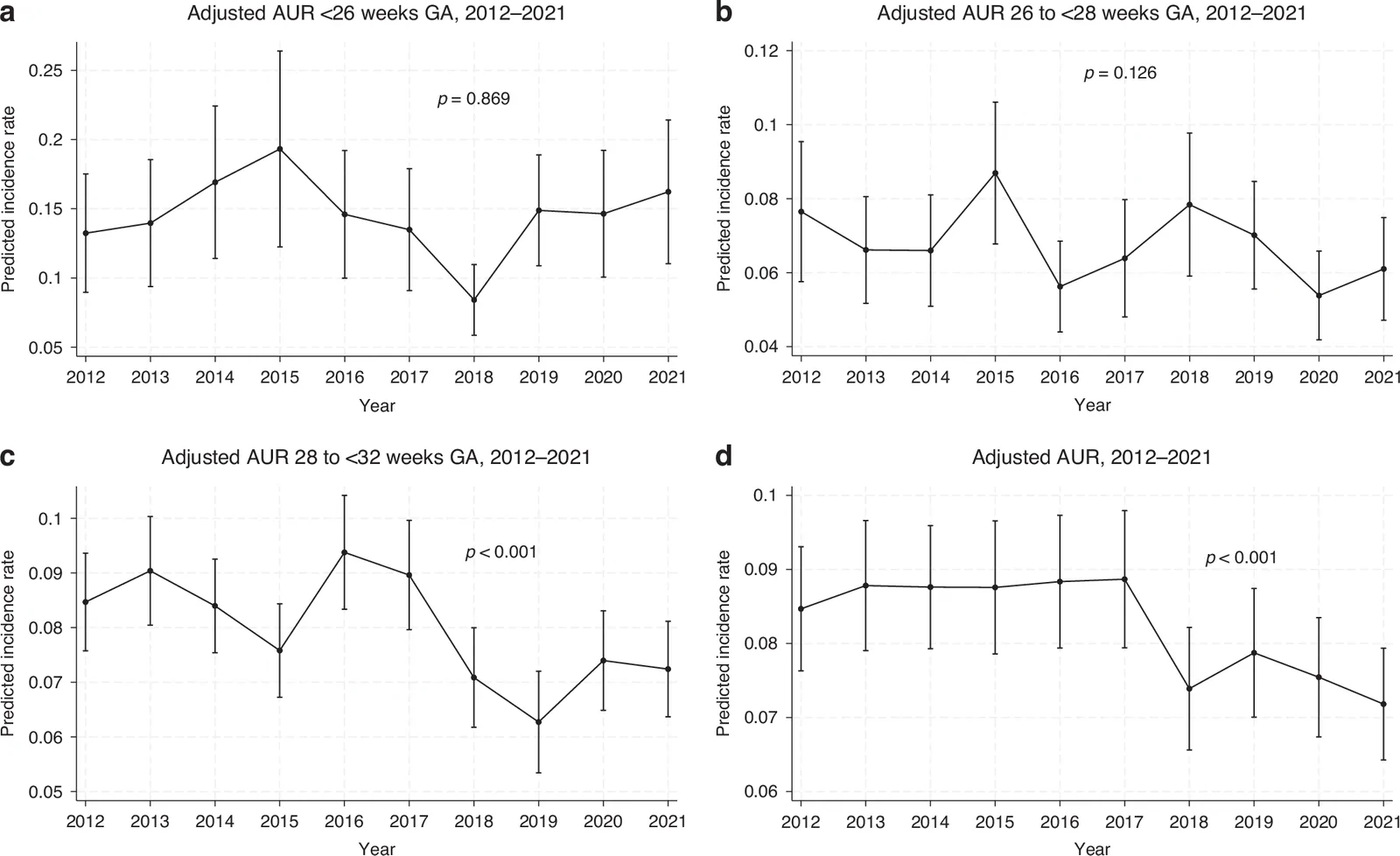
Temporal trends in antibiotic utilisation rate (AUR) in infants <32 weeks' gestational age (GA) without sepsis, necrotising enterocolitis (NEC) or spontaneous intestinal perforation (SIP). Line graphs of the change in AUR from 2012 to 2021 in (**a**) infants <26 weeks' GA, (**b**) infants 26 to <28 weeks' GA, (**c**) infants 28 to <32 weeks' GA and (**d**) all infants <32 weeks' GA without sepsis, NEC or SIP. Data are presented as estimated incidence rates (dots) and 95% confidence intervals (error bars) derived from binomial regression models adjusted for gestational age, birth weight z-score, sex and year of birth. Data for 2022 was incomplete and therefore excluded from this analysis.

**Fig. 3:**
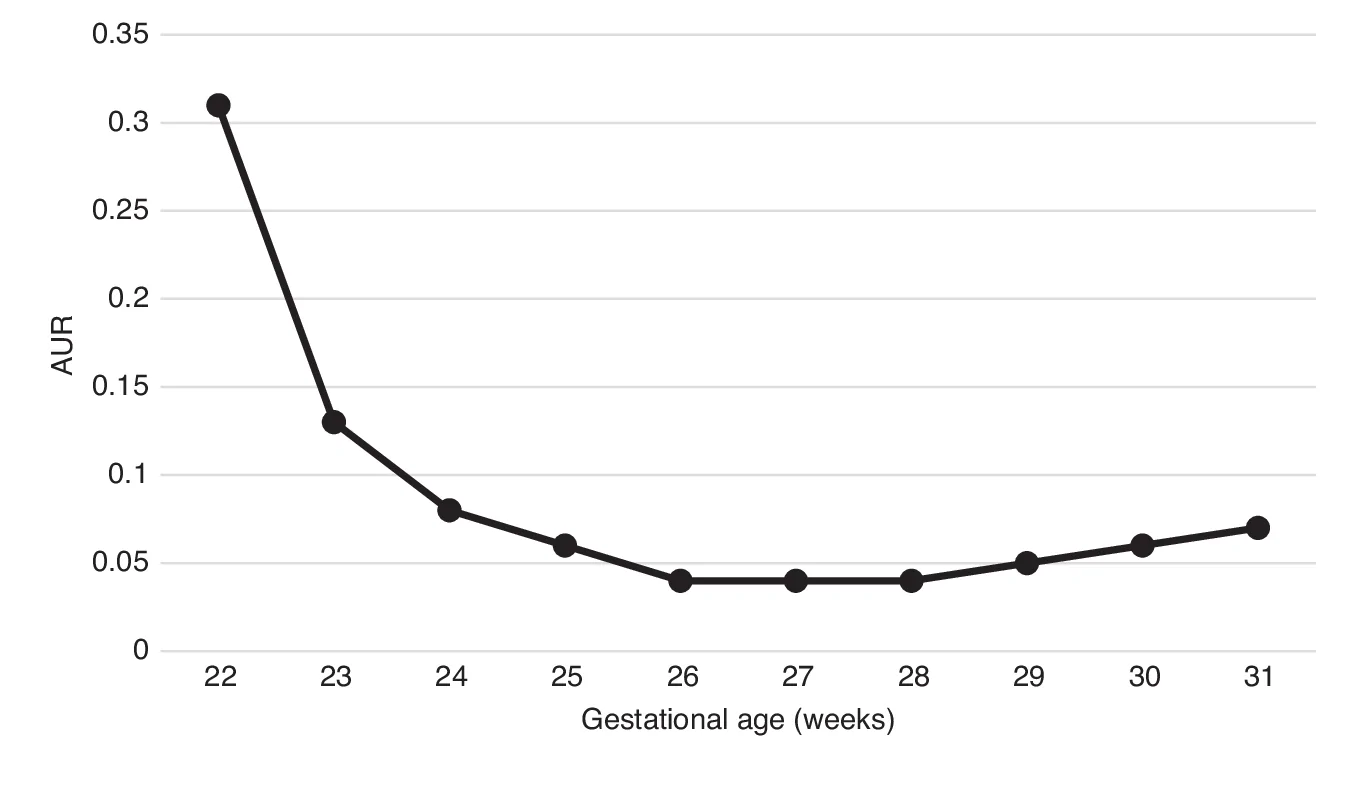
Antibiotic use by gestational age in infants <32 weeks' gestational age without sepsis, necrotising enterocolitis (NEC) and/or spontaneous intestinal perforation (SIP). Line graph of median antibiotic utilisation rate (AUR) by gestational age at birth in weeks.

DOT decreased from 78.0 to 61.9 per 1000 inpatient days and AUR from a median of 0.07 (IQR 0.04–0.11) to 0.05 (IQR 0.03–0.10) from 2012 to 2022 (Supplementary Table S1A). The mean estimate for AUR decreased for all infants <32 weeks combined from 0.08 (95% CI 0.07–0.09) to 0.07 (95% CI 0.06–0.07) (IRR 0.98, 95% CI 0.97–0.99; *p* < 0.001), and also for the 28 to <32-week gestational age group (*n* = 1967) from 0.08 (95% CI 0.07–0.09) to 0.07 (95% CI 0.06–0.08) (IRR 0.98, 95% CI 0.96–0.99, *p* < 0.001) from 2012 to 2021 (Fig. 2 and Supplementary Table S1B). There was no change in AUR for infants born <26 weeks (*n* = 398) and 26 to <28 weeks (*n* = 489) gestational age (Fig. 2 and Supplementary Table S1B). The proportion of infants <32 weeks receiving prolonged antibiotics decreased from 101 (35.7%) to 28 (21.1%) over the study period (adjusted IRR 0.94, 95% CI 0.91–0.96, *p* < 0.001) (Supplementary Table S1A). AUR was inversely proportional to gestational age with a median of 0.31 (IQR 0.07–1.00) at 22 weeks compared with 0.07 (IQR 0.05–0.12) at 31 weeks gestation (Fig. 3).

#### Neonatal outcomes associated with antibiotic exposure in infants without sepsis, NEC and/or SIP

**Fig. 4:**
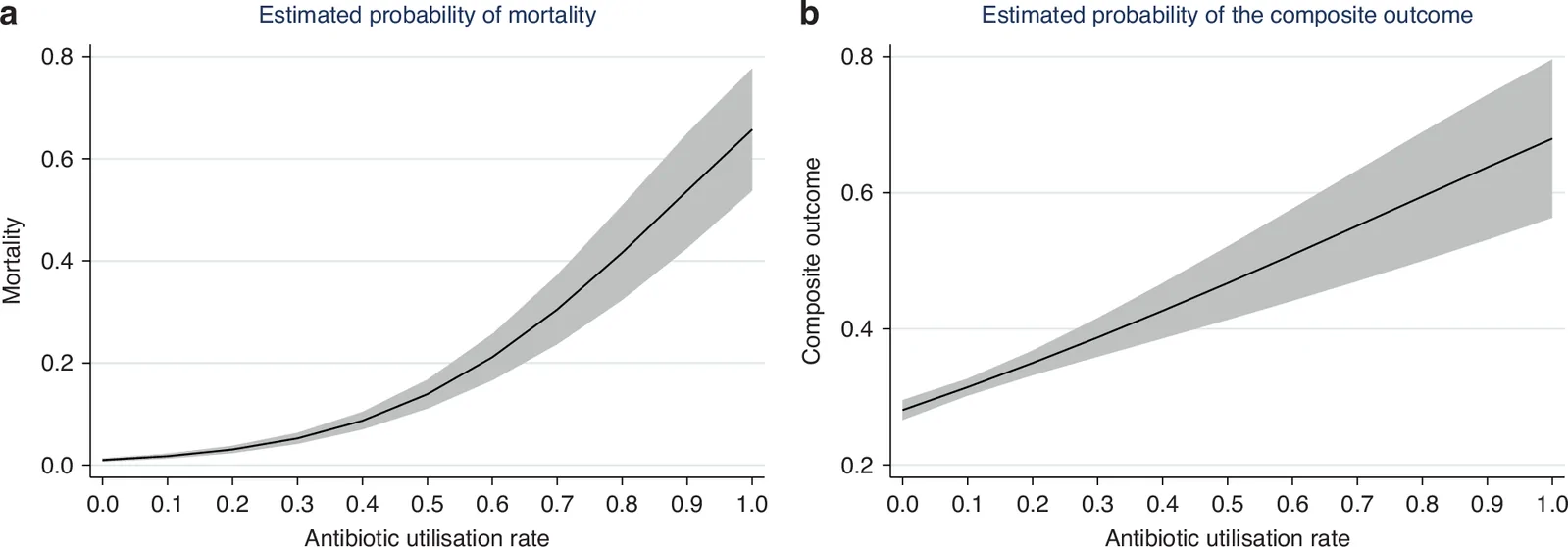
Estimated probability of adverse outcome according to the antibiotic utilisation rate (AUR). Line graph of estimated probability of mortality (**a**) and any composite outcome (**b**) according to AUR. The composite outcome included mortality and/or any major morbidity including late-onset sepsis, necrotising enterocolitis ≥ stage 2, chronic lung disease, severe retinopathy of prematurity and/or severe brain injury. Figures represent the predicted probabilities derived from logistic regression models adjusted for gestational age, birth weight z-score, sex and year of birth. The grey shaded area represents the 95% confidence interval for the predicted probability.

Higher AUR in CN infants was associated with increased mortality, significant brain injury, LOS, NEC, and the composite of any adverse outcome (Table 2A and Fig. 4). Using antibiotic duration ≤2 days as reference, antibiotic duration ≥5 days was associated with increased mortality, ROP, NEC, CLD, longer hospital stays, and the composite of any adverse outcome (Table 2B and Fig. 4). These findings were significant for ≥5 consecutive days of antibiotics but not for non-consecutive days for all outcomes except length of hospital stay (Supplementary Table S2).

**Table 2:** Mortality in infants who survived >12 hours and morbidity in infants surviving >7 days according to A) median antibiotic utilisation rate and B) duration of antibiotic exposure

A.				
	AUR (Median)	Unadjusted OR 95% CI	Adjusted OR 95% CI	*p*^*a*^
Mortality	0.67	1.97 (1.84–2.11)	1.81 (1.68–1.95)	< 0.001
Length of stay (days) (median, IQR)	-	0.92 (0.89–0.95)^β^	1.03 (1.00–1.06)^β^	0.057
LOS	0.24	1.65 (1.55–1.77)	1.52 (1.42–1.64)	< 0.001
NEC ≥Grade 2	0.17	1.39 (1.27–1.51)	1.22 (1.10–1.35)	< 0.001
CLD^b^	0.07	1.24 (1.17–1.32)	1.06 (0.97–1.16)	0.178
Severe ROP > Stage 3^c^	0.09	1.12 (1.02–1.22)	1.06 (0.99–1.13)	0.119
Significant brain injury	0.08	1.31 (1.22–1.41)	1.18 (1.09–1.29)	< 0.001
Any adverse outcomes^d^	0.08	1.43 (1.34–1.52)	1.32 (1.21–1.44)	< 0.001

B.				
	Antibiotic exposure ≥ 5 days	Unadjusted OR/HR 95% CI^e^	Adjusted OR/HR^*ae*^95% CI	*p*
Mortality	53 (6.3)	1.44 (0.99–2.10)	1.45 (1.19–1.76)	< 0.001
Length of stay (days) (median, IQR)	82 (53–114)	1.96 (1.77–2.18) ^β^	1.27 (1.15–1.41) ^β^	< 0.001
LOS	74 (8.9)	1.74 (1.25–2.41)	1.19 (1.00–1.43)	0.057
NEC ≥Grade 2	22 (2.7)	1.79 (0.99–3.23)	1.44 (1.10–1.89)	0.008
CLD^b^	406 (51.1)	7.88 (6.39–9.72)	2.54 (1.94–3.33)	< 0.001
Severe ROP > Stage 3^c^	101 (13.3)	7.90 (4.89–12.76)	1.95 (1.14–3.36)	0.015
Significant brain injury	59 (7.1)	3.38 (2.20–5.20)	1.21 (0.99–1.46)	0.058
Any adverse outcomes^d^	484 (58.4)	6.37 (5.27–7.70)	1.96 (1.52–2.52)	< 0.001

*AUR* antibiotic utilisation rate, *OR* odds ratio, *HR* hazard ratio, *CI* confidence interval, *LOS* late onset sepsis, *NEC* necrotising enterocolitis, *CLD* chronic lung disease, *ROP* retinopathy of prematurity; Significant brain injury: IVH ≥Grade 3 and/or cystic PVL, *β* Reported as hazards ratio.

^a^p-value represents the unadjusted and adjusted effects on outcomes of infants for a 10% increase in AUR. Adjustments were made for gestational age, birthweight z-score, sex and year of birth. Hazard ratios are interpreted as higher is worse (i.e.,longer duration of hospital stay has a higher hazard ratio).

^b^Excludes deaths before 36 weeks corrected gestational age.

^c^Assessed on infants <31 weeks gestation and/or birthweight <1250 g.

^d^Composite outcome of mortality, LOS, NEC, CLD, significant brain injury and severe ROP.

^e^Relative to antibiotics ≤2 days.

## Discussion

In this study, antibiotic exposure in infants <32 weeks’ GA without sepsis, NEC and/or SIP decreased from 2012 to 2022. However, antibiotic therapy for CN infants continued to occur. At the unit level (AUR), greater use aligned with higher odds of mortality, LOS, NEC and significant brain injury (composite likewise), with no adjusted association for CLD or severe ROP. At the patient level, longer courses were associated with higher odds of CLD and severe ROP, among other outcomes.

Several differences were noted in maternal and neonatal characteristics between infants with and without prolonged antibiotic exposure. Most of these parameters, including maternal chorioamnionitis, lower gestational age and birth weight, lower Apgar scores, mechanical ventilation and central lines, have been associated with higher risk of neonatal sepsis and may have resulted in reluctance to stop antibiotics despite negative cultures.^21–23^ Blood culture is considered the gold standard in diagnosis of neonatal sepsis but, despite poor performance characteristics, ancillary biomarkers are commonly used in addition and typically result in prolonged antibiotic exposure without evidence for improved outcomes.^22^

Most neonates in the study received antibiotics for suspected EOS based on obstetric risk factors and postnatal clinical presentation, but treatment was often continued despite sterile cultures.^24^ This prolonged exposure may be in response to elevated inflammatory markers and/or a clinically unwell baby with poor response to treatment. However, decreased use of unproven ancillary biomarkers can reduce antibiotic exposure without an increase in adverse outcomes in the first 7 days of life, and alternative diagnoses to sepsis should be considered in the unwell infant to reduce unnecessary antibiotic treatment.^25,26^ Recent approaches to the diagnosis and management of EOS in preterm infants, based on risk-stratification and de-implementation of biomarker use, are being increasingly recognized as both appropriate and safe.^24–26^ To avoid unnecessary antibiotic days, continuation decisions should prioritize cultures and clinical trajectory rather than CRP or other non‑specific biomarkers.

We observed a substantial reduction in antimicrobial exposure from 78.0 to 61.9 DOT per 1000 inpatient days corresponding with decreasing LOS incidence over the same period.^27^ The lower incidence of LOS was most likely related to implementation of quality improvement strategies during the study period including early introduction and faster advancement of enteral feeds, topical coconut oil for skin integrity, decreased central lines, improvements in venous line insertion and maintenance, and hand hygiene.^27^ A greater decrease in antibiotic consumption from 100 to 40 DOT per 1000 inpatient days has been reported in infants <32 weeks in Norway, highlighting that further progress is feasible.^4^ Future opportunities include improvement of antimicrobial stewardship (AMS) interventions, improving blood culture technique to reduce false positives, optimising inoculum volume to increase yield, prompt discontinuation of antibiotics when cultures are negative, and identification of novel biomarkers with a high negative predictive value.^28–30^

We found higher odds of adverse neonatal outcomes associated with longer antibiotic exposure in CN infants. Higher AUR and/or prolonged exposure were associated with increased mortality, brain injury, NEC, LOS, CLD, longer hospital stays, and the composite of any adverse outcome, with a dose-dependent effect. This association is well described and likely related to disruption of the neonatal microbiome.^6,7,31–35^ Colonization with commensal organisms is protective, preventing colonization with pathogenic organisms which can subsequently cause sepsis and/or NEC.^7,36^ Mucosal microbiota critical for instruction of the developing immune system effect immunological alterations in the gut, development of immune tolerance and competence, and inflammatory balance.^7,36,37^ Although the neonatal microbiome may recover following shorter courses of antimicrobials, prolonged exposure has been associated with permanent perturbations of the microbiome and long term health effects including obesity, asthma, atopy, inflammatory bowel disease, diabetes and multiple sclerosis.^7,10,36,38^

The adverse effects of prolonged exposure had a stronger association with consecutive rather than non-consecutive antibiotic days. Although any antibiotic exposure disrupts the intestinal microbiome, longer exposure is associated with extended dysbiosis, slower recovery and greater antimicrobial resistance. It is possible that short, non-consecutive courses of antibiotics give the intestinal microbiome a chance to recover between treatment episodes, resulting in less detrimental effect, emphasizing the importance of timely cessation of antibiotic therapy.^36,39^ Considering >90% of blood cultures are positive by 36 h, this would be a reasonable time point to stop antibiotics in CN infants to reduce unnecessary antibiotic exposure.^40–42^

This study had several strengths, including the large sample size of a contemporaneous cohort, data extraction from a well-established electronic database, and rigorous data analysis. However, the study also had limitations inherent to its monocentric and retrospective nature which limit generalizability. There was also the potential for confounding by indication, with sicker infants receiving longer courses of antibiotics, despite statistical modelling. Inclusion of laboratory data, such as ancillary biomarkers, and an estimate of illness severity, may have added value but were beyond the scope of the study.

In conclusion, antibiotics are critical therapy for infants with sepsis but can cause harm in those without. Potential future strategies to reduce antibiotic exposure include implementing evidence-based antimicrobial stewardship programs, identification of and withholding antibiotics in infants at low infection risk, optimization of blood culture collection technique and volume, and identification of novel biomarkers with high predictive values.^30,43^

## Supplementary information

Supplementary Table 1

Supplementary Table 2

## Data Availability

All data generated or analysed during this study are included in this article. Further enquiries can be directed to the corresponding author.
