# Supplementary material for: Antibiotic exposure in culture-negative preterm infants: a 10-year single-centre study
Source: Pediatr Res. 2026 Jan 10;100(3):1324–32. doi: 10.1038/s41390-025-04707-5 (PMC13672043; doi:10.1038/s41390-025-04707-5)
Supplement: Supplementary file 1 [file 41390_2025_4707_MOESM1_ESM.pdf]

Supplementary Table S1(A): Temporal trends in antibiotic use in infants <32 weeks' gestation without culture-confirmed sepsis, NEC and/or SIP

	2012	2013	2014	2015	2016	2017	2018	2019	2020	2021	2022 [a]	Total
**Number of patients**	283	276	306	279	312	286	259	269	290	275	133	2968
**Inpatient days**	17069	16227	17918	16115	17453	16719	14993	16600	16961	16753	7624	174429
**Total antibiotic days**	1332	1331	1460	1282	1218	1214	921	1102	1064	1056	472	12452
**DOT**[b]	78.0	82.0	81.5	79.6	69.8	72.6	61.4	66.4	62.7	63.0	61.9	71.4
**AUR (median, IQR)** [c]	0.07 (0.04-0.11)	0.07 (0.04-0.12)	0.06 (0.04-0.11)	0.06 (0.04-0.10)	0.05 (0.03-0.09)	0.05 (0.03-0.10)	0.05 (0.03-0.08)	0.04 (0.02-0.08)	0.05 (0.03-0.09)	0.04 (0.03-0.08)	0.05 (0.03-0.10)	0.05 (0.03-0.10)
**Any antibiotics (n, %)**	274 (96.8)	266 (96.4)	295 (96.4)	255 (91.4)	272 (87.2)	248 (86.7)	227 (87.6)	231 (85.9)	259 (89.3)	254 (92.4)	121 (91.0)	2702 (91.0)
**Prolonged antibiotics (n, %)**[d]	101 (35.7)	93 (33.7)	99 (32.4)	66 (23.7)	65 (20.8)	76 (26.6)	51 (19.7)	60 (22.3)	57 (19.7)	53 (19.3)	28 (21.1)	749 (25.2)
**Antibiotic days per patient (median, IQR)**	3 (2-6)	3 (2-5)	3 (2-5)	3 (2-4)	2 (2-4)	2 (2-5)	2 (2-4)	2 (2-4)	2 (2-4)	2 (2-4)	2 (2-4)	2 (2-5)
**Suspected EOS (n, %)**	272 (96.1)	265 (96.0)	293 (95.8)	249 (89.2)	268 (85.9)	242 (84.6)	224 (86.5)	224 (83.3)	253 (87.2)	246 (89.5)	118 (88.7)	2654 (89.4)
**Suspected LOS (n, %)**	74 (26.1)	63 (22.8)	84 (27.5)	62 (22.2)	54 (17.3)	60 (21.0)	51 (19.7)	68 (25.3)	73 (25.2)	69 (25.1)	31 (23.3)	689 (23.2)
**Mortality (n, %)**	14 (4.9)	9 (3.3)	13 (4.2)	15 (5.4)	15 (4.8)	11 (3.8)	8 (3.1)	15 (5.6)	15 (5.2)	9 (3.3)	8 (6.0)	132 (4.4)

DOT: Days of treatment per 1000 inpatient days; AUR: antibiotic utilisation rate; IQR: interquartile range; EOS: early onset sepsis; LOS: late onset sepsis; NEC: necrotising enterocolitis.

[a] Represents data up to 30 June 2022; [b]Defined as total antibiotic days per 1000 inpatient days; DOT decreased from 2012 to 2022 (IRR 0.97, 95% CI 0.96-0.98, p<0.001); [c]Defined as the number of days an infant was exposed to 1 or more antimicrobial agents divided by the total length of hospital stay; AUR decreased from 2012 to 2022 (IRR 0.98, 95% CI 0.97-0.99, *p*<0.001); [d]Defined as antibiotic treatment ≥5 days.

Supplementary Table S1(B): Temporal trends in antibiotic utilisation rate (AUR) in infants without culture-confirmed sepsis, NEC and/or SIP by gestational age[a]

	**2012**	**2013**	**2014**	**2015**	**2016**	**2017**	**2018**	**2019**	**2020**	**2021**	***p***
	n=283	n=276	n=306	n=279	n=312	n=286	n=259	n=269	n=290	n=275	
**Gestational age (w)**											
**<26 (n=398)**	0.13 (0.09-0.18)	0.14 (0.09-0.19)	0.17 (0.11-0.23)	0.18 (0.12-0.25)	0.15 (0.10-0.20)	0.14 (0.09-0.18)	0.08 (0.06-0.11)	0.15 (0.11-0.19)	0.15 (0.10-0.19)	0.16 (0.11-0.21)	0.814
**26 to <28 (n=489)**	0.08 (0.06-0.10)	0.07 (0.05-0.08)	0.07 (0.05-0.08)	0.09 (0.07-0.10)	0.06 (0.04-0.07)	0.06 (0.05-0.08)	0.08 (0.06-0.10)	0.07 (0.06-0.08)	0.05 (0.04-0.07)	0.06 (0.04-0.07)	0.054
**28 to <32 (n=1967)**	0.08 (0.07-0.09)	0.09 (0.08-0.10)	0.08 (0.07-0.09)	0.07 (0.06-0.08)	0.08 (0.07-0.09)	0.07 (0.07-0.08)	0.06 (0.05-0.07)	0.05 (0.04-0.06)	0.06 (0.05-0.07)	0.07 (0.06-0.08)	<0.001
**All (n=2835)**	0.08 (0.07-0.09)	0.09 (0.08-0.10)	0.09 (0.08-0.10)	0.08 (0.07-0.09)	0.08 (0.07-0.09)	0.08 (0.07-0.09)	0.07 (0.06-0.07)	0.07 (0.06-0.07)	0.07 (0.06-0.08)	0.07 (0.06-0.07)	<0.001

[a] Data presented as estimated incidence rates and 95% confidence intervals derived from negative binomial regression models adjusted for gestational age, birth weight z-score, sex and year of birth. Data for 2022 was incomplete and therefore excluded from this analysis.
