# Supplementary material for: Antibiotic exposure in culture-negative preterm infants: a 10-year single-centre study
Source: Pediatr Res. 2026 Jan 10;100(3):1324–32. doi: 10.1038/s41390-025-04707-5 (PMC13672043; doi:10.1038/s41390-025-04707-5)
Supplement: Supplementary file 2 [file 41390_2025_4707_MOESM2_ESM.pdf]

Supplementary Table S2: Outcomes following consecutive vs non-consecutive antibiotics for ≥5 days

	Antibiotic exposure (days)	N (%)	Unadjusted OR/HR 95% CI	Adjusted OR/HR[a] 95% CI	*p*
Mortality	**<=2**	77 (4.8)	1.00	1.00	
	≥5 non-consec	11 (5.0)	1.02 (0.54-1.96)	1.38 (0.96-1.99)	0.080
	≥5 consec	42 (6.8)	1.43 (0.97-2.11)	1.48 (1.20-1.82)	<0.001
Length of stay (days) (median, 25th-75th %ile)	**<=2**	48 (35-65)	HR:1.00	HR:1.00	
	≥5 non-consec	81 (58-111)	HR: 3.00 (2.70-3.33)	HR: 1.08 (1.03-1.13)	0.002
	≥5 consec	82 (52-115)	HR: 1.08 (0.92-1.26)	HR: 0.94 (0.88-1.01)	0.068
LOS	**<=2**	82 (5.3)	1.00	1.00	
	≥5 non-consec	18 (8.1)	1.57 (0.92-2.67)	1.17 (0.87-1.58)	0.311
	≥5 consec	56 (9.2)	1.80 (1.26-2.56)	1.20 (0.99-1.46)	0.065
NEC ≥Grade 2	**<=2**	23 (1.5)	1.00	1.00	
	≥5 non-consec	5 (2.3)	1.52 (0.57-4.04)	1.05 (0.60-1.84)	0.866
	≥5 consec	17 (2.8)	1.89 (1.00-3.56)	1.56 (1.18-2.08)	0.002
CLD[b]	**<=2**	177 (11.7)	1.00	1.00	
	≥5 non-consec	106 (49.5)	7.41 (5.44-10.11)	2.64 (1.77-3.93)	<0.001
	≥5 consec	300 (51.6)	8.06 (6.43-10.11)	2.50 (1.85-3.37)	<0.001
Severe ROP > Stage 3[c]	**<=2**	21 (1.9)	1.00	1.00	
	≥5 non-consec	23 (11.6)	6.70 (3.63-12.37)	1.97 (1.00-3.89)	0.050
	≥5 consec	78 (14.0)	8.33 (5.09-13.65)	2.09 (1.19-3.69)	0.011
Significant brain injury	**<=2**	34 (2.2)	1.00	1.00	
	≥5 non-consec	13 (5.9)	2.76 (1.43-5.31)	1.48 (0.73-3.02)	0.280
	≥5 consec	46 (7.6)	3.61 (2.29-5.69)	2.02 (1.18-3.45)	0.011
Any adverse outcomes[d]	**<=2**	277 (18.1)	1.00	1.00	
	≥5 non-consec	125 (56.6)	5.91 (4.39-7.95)	1.97 (1.34-2.90)	<0.001
	≥5 consec	359 (59.0)	6.54 (5.32-8.05)	2.01 (1.52-2.66)	<0.001

OR: odds ratio; HR: hazard ratio; CI: confidence interval; LOS: late onset sepsis; NEC: necrotising enterocolitis; CLD: chronic lung disease, ROP: retinopathy of prematurity, Significant brain injury: IVH ≥Grade 3 and/or cystic PVL. β: Reported as hazards ratio.

[a]Adjustments were made for gestational age, birthweight z-score, sex and year of birth. Hazard ratios are interpreted as higher is worse (i.e.,longer duration of hospital stay has a higher hazard ratio).

[b]Excludes deaths before 36 weeks corrected gestational age

[c]Assessed on infants <31 weeks gestation and/or birthweight <1250g

[d]Composite outcome of mortality, LOS, NEC, CLD, significant brain injury and severe ROP.
